# Simulated drought stress unravels differential response and different mechanisms of drought tolerance in newly developed tropical field corn inbreds

**DOI:** 10.1371/journal.pone.0283528

**Published:** 2023-03-27

**Authors:** Naidu Gopalakrishna K., Rajeshwari Hugar, Kachapur Rajashekar M., Bhat Jayant S., Sidramappa C. Talekar, Chimmad Virupaxi P.

**Affiliations:** 1 AICRP on Maize, University of Agricultural Sciences, Dharwad, Karnataka, India; 2 Department of Genetics and Plant Breeding, College of Agriculture, University of Agricultural Sciences, Dharwad, Karnataka, India; 3 Indian Agriculture Research Institute, Regional Research Centre, Dharwad, Karnataka, India; 4 Department of Crop Physiology, University of Agricultural Sciences, Dharwad, Karnataka, India; University of the West Indies, TRINIDAD AND TOBAGO

## Abstract

Corn is one of the most important cereal crops in the world with highest yield potential. Nevertheless, its potential productivity is constrained by the occurrences of drought stress worldwide. Besides, in the era of climate change, frequent occurrences of severe droughts are predicted. The present investigation was carried out at Main Agricultural Research Station, University of Agricultural Sciences, Dharwad in split plot design to study response of twenty-eight new corn inbreds under drought free (well-watered) conditions and drought simulated by withholding irrigation from 40 to 75 DAS to create water stress. Significant differences among the corn inbreds, moisture treatments and interaction between inbreds were observed for morpho-physiological, yield and yield components indicating differential response of corn inbreds. The inbreds CAL 1426–2 (higher RWC, SLW& wax and lower ASI), PDM 4641(higher SLW, proline, & wax, and lower ASI) and GPM 114 (higher proline & wax, and lower ASI) were drought tolerant. These inbreds are having higher production potential (>5.0 t/ha) under moisture stress condition with less per cent reduction (<24.4%) over non-moisture stress condition and hence are putative candidates for developing drought tolerant hybrids suitable for rainfed ecosystem besides using them in population improvement program to combine different drought tolerant mechanisms to evolve highly potent drought tolerant inbreds. The results of the study suggested that proline content, wax content, anthesis silking interval, relative water content can be better surrogate traits to identify drought tolerant inbreds in corn.

## Introduction

Corn (*Zea mays* L.), is grown world-wide due to its high production potential. In India, the corn production has increased from 1.7 Mt during 1950–51 to 28.2 Mt during 2018–19 and productivity increased from mere 547 kgha^-1^ to 3530 kgha^-1^ in the corresponding period. There is ever increasing demand for corn due to its multiple uses which may exceed 500 Mt in the developing countries and may surpass the demand of wheat and rice by the year 2025 [1]. However, the corn production is affected greatly by various biotic and abiotic stresses during the growing season. Among these, drought stress is an important constraint to fulfill the increasing demand. This is even more exacerbated by the ongoing climate change featuring greater intensity and frequent droughts [2] resulting in negative impact on yield of different crops.

Corn, more suited to irrigated eco-system, will be adversely affected by climatic changes and its yields would be reduced by drought by affecting length of the growing season [3]. As compared to normal irrigation, there is significant decrease in grain yield under mild (80.1%) and severe drought (93.6%). Drought occurring at vegetative stage followed by tasselling and silking of corn results in maximum yield loss [4, 5]. Likewise drought stress during tasseling can lead to yield reduction to an extent of 22% [6]. Different management practices that aim to reduce water loss have been recommended to maximize yield under drought. These practices include agronomic measures (mulching, wider row spacing; [7]), physiological treatments (use of growth regulators; [8]) and use of drought tolerant cultivars [9].

The term drought tolerance in the plant breeder’s perspective can be defined as “the ability of a crop to maintain its biomass production during drought conditions” [10]. The drought tolerance is conditioned by different mechanisms *viz*., maintaining relative water content, specific leaf area, chlorophyll content, shorter anthesis silking interval, increased proline and wax content to combat drought situation. Hence, the adaptations/mechanisms that promote retention of water in the plants and higher seed set under moisture stress may result in drought tolerance.

Breeding for drought tolerance in corn is challenging due to its complex inheritance compounded with G x E interaction and confounding soil factors. In the development of drought tolerant hybrids, identification of drought tolerant inbreds and understanding the mechanisms contributing to drought tolerance are important preliminary steps. A study of the drought tolerance levels of six inbred lines and four hybrids indicated five secondary traits (RWC, leaf rolling, leaf senescence, ASI, ears/plant) were effective indicators for the selection of drought tolerant corn genotypes [11, 12]. opined that high RWC in the inbreds is closely related to drought tolerance [13]. while evaluating inbred lines for drought tolerance indicated that, anthesis-silking interval, leaf relative water content, stomatal count, chlorophyll content before flowering, chlorophyll content before maturity, ears per plant, grain yield per plot, protein content were to be given more weightage while applying selection for improvement of these traits and in identifying drought tolerant lines [14]. studied 35 corn inbreds under field condition by withholding irrigation before 10 days of flowering and stopped for about one month and the irrigation was resumed when soil moisture reached permanent wilting point at a depth of 40–60 cm. Nine inbreds showed high tolerance to drought by maintaining shorter ASI, higher shelling %, moisture %, ear length, ear diameter, 100 grain weight and comparatively higher grain yield under drought stress. The ASI is known to have a strong relationship with grain yield due to increased pollination and seed set when the period between anthesis and silking is short. Hence, narrower ASI is more important especially under drought conditions [15]. Proline accumulation showed positive correlation with drought stress [16–18]. [19] reported the effects of stress on proline accumulation in corn variety and interaction was found significant. The study on the drought tolerance in 12 corn hybrids observed that increased proline content and wax content in the drought tolerance corn hybrids under moisture stress and suggested to use these parameters as most reliable parameters for the phenotypic drought tolerant screening [20]. Therefore, the drought tolerant cultivars in corn can be developed by the introgression of these mechanisms in the elite inbreds and combining them in hybrids cultivars through genetical approaches. Successful hybrids developed using the drought tolerant inbreds which performed better under moisture stress situations [21–23].

The development of corn hybrids with enhanced tolerance to drought stress and higher water use efficiency (WUE) has become a high priority for major breeding programs, both in the private and public levels. This is important for any corn production regions in general and in India, particularly, as majority of corn production areas is still rainfed and expected to face vagaries of climate change. Understanding the nature of drought response and drought tolerance mechanisms in the corn genotypes would provide opportunities to improve the breeding process and to device suitable breeding strategies in developing drought tolerant corn [15].

Drought tolerance can be assessed only by comparing the performance of breeding lines under water stress and non-stress (irrigated) conditions. Using the data from these two moisture regimes, breeders can calculate drought intensity index for the experiment and the different susceptibility indices and means to assist in selection of drought tolerant genotypes [24]. Alternatively, yield improvement in water limited environments could be achieved by identifying secondary traits, such as relative water content, proline content, contributing to drought tolerance and selecting for those traits in a breeding program. Thus, the current study was planned to delineate the response of corn inbreds to mid-season drought stress for various physiological, yield and yield components under well-watered and water-stressed environments. This in turn helps in identifying drought tolerant corn inbreds and understanding different physiological mechanisms contributing to drought tolerance and to devise suitable breeding strategies in relation to the inbreds studied.

## Materials and methods

The study included twenty-eight diverse corn inbred lines, collected from All India Coordinated Research Project on Maize, University of Agricultural Sciences, Dharwad and Indian Agricultural Research Institute, Regional Research Centre, Dharwad (Table 1). These inbreds were developed from diverse background to develop hybrids suitable for rainfed conditions and they were selected in the normal rainfed conditions during their development and hence were included for studying their reaction for moisture stress under field condition. The study was conducted during post-rainy season of 2020–21 at All India Coordinated Research Project on Maize, Main Agricultural Research Station, University of Agricultural Sciences, Dharwad (15°26′N latitude, 70°26′E longitude and 678 m above the mean sea level). Post-rainy season was chosen for the study to ensure rain free period during drought simulation. Detailed weather parameters during the experiment are provided in Table 2. The soil in the experimental plot was medium deep black (*Vertic Inseptisol*).

**Table 1:** List of corn genotypes with their pedigree and source.

Sl. No.	Genotype	Pedigree	Source
1	PDM 77–4	(Comp 85164 × Comp 8527) × 10-2-8-7-1-1-4-f	IARI Regional Research center, Dharwad
2	PDM 260–1	PS-28-3-1-2-2-1-1-AE	
3	PDM 4341	(Comp8551 X Comp 8527 x Ageti 76 X MDR) -9- 4-2-8-7-1-1-2-1-L-1	
4	PDM 4251	PS-25-1-1-1-1-1-1-1-1-R-1	
5	PDM 4641	KDMH-176-5-1-1-R-1	
6	PML 17	KDMH-176-5-1-1-R-2	
7	PML 46	SAFAL-X12-9-1-1	
8	PML 93	KDMH-176-5-1-1-R-6-1	
9	PML 54	KDMH-755-12-1-1	
10	PML 102	KMH-218PLUS-1-1-3-R-1	
11	DIM 204	Advanta 7074-1-2-1-1-1	
12	DIM 302	PHB-12-1-3-3-1-K-1	
13	CDM 112	CA 1 45 14–1 0-8-2-8*4–8	
14	D 2287	PMH-3-2Bulk-Bulk-1-2-1-1	
15	D 1013	Sel-LCY3-7-1-2-2-1-1-f	
16	CM 111	Cuba-342-2-F-#-#	AICRP on Maize, UAS, Dharwad
17	GPM 114	EC 618990	
18	CML 451	Hy 09R-N9251-18	
19	CAL 1426–2	CA-1457/P145C4MH7-1-B-1-1-B-1-1B*17	CIMMYT, Hyderabad
20	CML 563	HY18 R-Y75-2	
21	IMIC 2030	VL-19008-[DTPYsyn16HG(B)]-6-2-1-2-B_1_	
22	CML 579–1	HY 18 R-Y75-6-1	
23	CML 579–2	HY18R-Y75-6-2	
24	CML 580	HY18R-Y75-7	
25	CML 582	(CA-34505 × CA-00302)-B-2-1-B-1-BB(T-B3-#15-2-B-1-B*6-B2)	
26	IMIC 2024	VL-162283-AMDROUT1c3-B-5-1-BB-B_1_	
27	PML 9	Polo-1-2-2-R-1-R-1-2	IARI Regional Research center, Dharwad
28	PML 21	DMH-119-1-1-4-K-1-K-1-21	

**Table 2:** Weather conditions during the crop period (December 2020 to May 2021) at Main Agriculture Research Station, UAS, Dharwad, Karnataka, India.

Month	Rainfall (mm)		Rainy days	Temperature (°C)				Relative humidity (%)	
	1950–2020	2021							
				Maximum		Minimum		1950–2020	2021
				1950–2020	2021	1950–2020	2021		
December	0.4	0	0	28.5	28.9	14.2	14.6	60.9	75.4
January	0.8	27.2	3	29.5	29.4	14.7	15.9	54.9	63.4
February	10.7	10.0	1	32.0	30.3	16.4	15.2	45.9	52.8
March	9.7	0.4	0	34.6	34.8	19.1	18.5	45.6	44.5
April	40.6	103.0	9	36.3	35.4	20.9	20.5	56.8	58.5
May	43.7	129.2	14	36.3	31.7	22.0	21.4	62.8	70.4
Mean	17.6	45.2	4.5	32.9	31.8	17.9	17.7	54.6	61.0

The inbreds were sown on 14^th^ December 2020 in a split plot design consisted of two replications with water treatments as main factor and genotypes as sub factor. In each replication, each inbred was hand dibbled into two rows of 4 m row length with a spacing of 60 cm wide and 20 cm plant spacing so as to have at least 37 plants in the plot.

**Fig 1:**
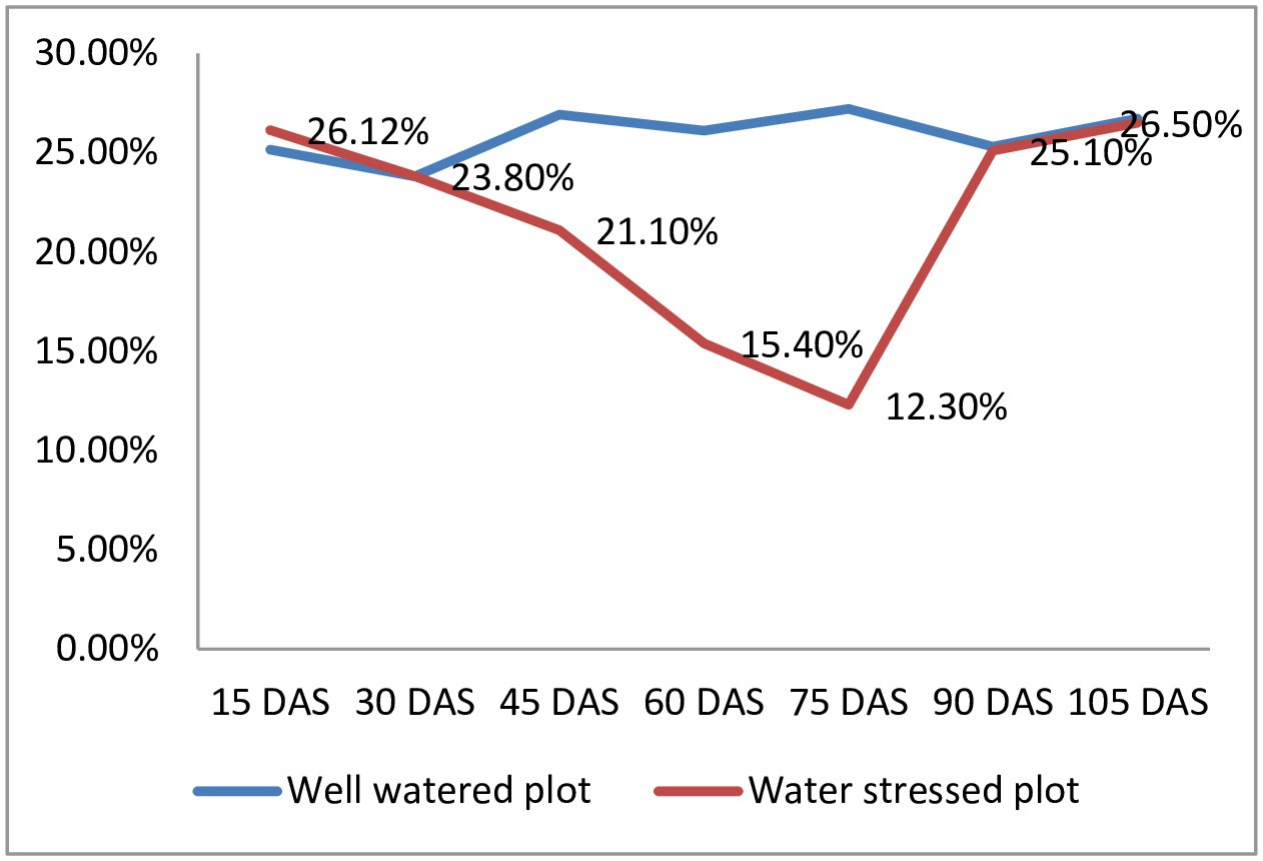
Moisture status in the experimental field.

Check basin method of surface irrigation was used to irrigate each plot initially for a depth of 3 cm followed by to a depth of 5 cm. Parshall flume was installed in the field to measure the quantum of irrigation water. Irrigation schedule was followed same in both well watered and water stressed plots, at an interval of 10–12 days until 40 days after sowing (DAS). Thereafter, the scheduled irrigation was continued in well-watered plots while, irrigation was withheld in water stressed plots until 75 DAS. This corresponded to 568 °C to 785 °C growing degree day units (GDDU) on 40 DAS and 75 DAS, respectively [25]. This water stress period of 35 days was coinciding with the pre-tasselling to initiation of seed formation. The irrigation schedule was restored on 76 DAS in water stressed plots and subsequently, the same irrigation schedule was followed in both moisture regimes. A buffer area of 2 m (with a trench) was set between the well watered and water stress plots to prevent the lateral movement of irrigation water into water stress plots. Other recommended agronomic practices like hoeing between rows at 25 and 45 DAS and weeding at 60 and 80 DAS to control weeds, and plant protection measures such as spraying emamectin benzoate 5% SG and chlorantriniliprole 18.5% w/w insecticide at 20 and 35 DAS, respectively to control fall army worm, were followed. The soil moisture level was measured randomly from five spots separately in each replication under both well watered and water stressed plots at 15 days’ interval and the soil moisture details are provided in Fig 1. Growing degree days (GDD) was calculated using the following formula with 10 °C as the base temperature for corn [26].

$$\text{GDD}=\Sigma \frac{{\text{T}}_{\text{max}}+{\text{T}}_{\text{min}}}{2}-{\text{T}}_{\text{b}}$$

Where,

T_max_–Daily maximum temperature

T_min_–Daily minimum temperature

T_b_–Base temperature (10 °C for corn)

Five plants from each plot were randomly selected and tagged from both the rows (2 or 3 plants in each row) to account for row effect at 30 DAS for recording morpho-physiological observations. Besides, pollen fertility at 50% tasseling, days to 50% tasseling and days to 50% silking were also recorded. Anthesis-silking interval was calculated as the difference between the days to 50% tasselling and days to 50% silking. Plant height and ear height (position level of the ear on the stem from the ground) were recorded at the time of harvest in five randomly tagged plants. Post harvest observations *viz*., ear length, ear diameter, kernel row number, number of kernels per row were measured from five plants which were earlier selected randomly and tagged. Ear diameter was measured using vernier calliper on five ears from the same five plants which have been tagged for taking various morphological observations. Shelling percentage and 100 seed weight were recorded from randomly selected and tagged five plants, which were used to record other ear observations. Shelling percentage is measured as the ratio of kernels to the ear weight and expressed in percentage. The grain yield was recorded from entire plot harvest. Harvest index was calculated as the ratio of grain yield to the total biomass at each plot level.

### Relative leaf water content (RWC)

Relative water content was estimated by following the procedure outlined by [27] at 60 and 75 DAS. Ten leaf discs of 10 mm were collected in the middle of 3^rd^ fully expanded leaf from the top and weighed using electronic balance and fresh weight in gram was recorded. The weighed leaf discs were floated in a petri-dish containing distilled water for four hours and subsequently blotted gently and weight was taken again, which was referred to as the turgid weight. After taking turgid weight, the leaf discs were oven dried at 80°C for 48 hours and dry weight was recorded. The RWC was calculated using the following formula and expressed in percentage.


$$\text{Relative}\;\text{leaf}\;\text{water}\;\text{content}=\frac{\text{Fresh}\;\text{weight}\;\left(\text{g}\right)–\text{Dry}\;\text{weight}\;\left(\text{g}\right)}{\text{Turgid}\;\text{weight}\;\left(\text{g}\right)–\text{Dry}\;\text{weight}\;\left(\text{g}\right)}\times 100$$

### Specific leaf weight (SLW)

Ten leaf discs were collected at 60 and 75 DAS and oven dried at 80 °C until constant weight is achieved and leaf dry weight was calculated. The leaf area was determined by using formula *πr*^2^. The SLW was calculated by using the following formula.


$$\text{SLW}\;\left(\text{g}/{\text{dm}}^{2}\right)=\frac{\text{Leaf}\;\text{dry}\;\text{weight}\;\left(\text{g}\right)}{\text{Leaf}\;\text{area}\;\left({\text{dm}}^{2}\right)}$$

### SPAD Chlorophyll meter reading (SCMR)

The chlorophyll meter SPAD 502 (Soil Plant Analysis Development meter, Konica Minolta) measures the absorbance of the leaf in the red and near infrared region. Using these two transmittances, it calculates a numerical SPAD value which is proportional to the chlorophyll present in the leaf and is negatively related to chlorosis of the plants. SCMR was taken at 60 and 75 DAS. Top, middle and bottom leaves were used for measuring SPAD, which was taken on one side of leaf blade, midway between the leaf base and tip. The reading was taken between 10.00 and 12.00 hrs of the day. In each plant, there will be three readings (top, middle and bottom) and mean of three will be taken for five plant mean calculation. A mean of five readings per inbred was taken from five tagged plants.

### Pollen fertility

Fresh pollen was collected between 9.00 to 10.00 am from tassel of five random plants for each inbred in a tassel bag and pollen fertility was assessed using acetocarmine stain. Those which have taken the stain are referred to as fertile pollen and the unstained ones are considered as non-fertile. Total number of pollen grains was counted from the average of three microscopic areas. Pollen fertility was calculated using the following formula

$$\text{Pollen}\;\text{fertility}=\frac{\text{Number}\;\text{of}\;\text{fertile}\;\text{pollen}\;\text{grains}}{\text{Total}\;\text{number}\;\text{of}\;\text{pollen}\;\text{grains}}\times 100$$

### Proline content

Proline content was estimated at 75 DAS by the procedure described by [28]. Plant tissue in the form of 10 leaf discs (0.5 g) was taken from middle leaf of five plants in each inbred and homogenized in 5 ml of 3% sulpho-salicylic acid, which was centrifuged at 3000 rpm and supernatant was collected for estimation of proline content. After this 2 ml of filtrate was taken and added with 2 ml of glacial acetic acid and 2 ml of ninhydrin reagent followed by heating the reaction mixture in a water bath at 100 °C, for about 1 hour, until brick red colour was developed. Then the reaction mixture was cooled, to which 4 ml of toluene was added and then transferred to a separating funnel. After thorough mixing, the chromospheres containing toluene was separated. This is proline sample mixture and standard curve of proline was prepared by taking 5 to 100 μgml^-1^ concentration with absorption readings at 520 nm in spectrophotometer (model: Elico BL 222, Double beam). The free proline content in samples was estimated by referring to a standard curve prepared from known concentrations of proline. The proline content in leaf tissue was calculated by using the formula

$$\text{Proline}\;(\text{mol}/\text{g}\;\text{fresh}\;\text{weight})\;=\frac{3.11\times \text{OD}520\times \text{V}}{\begin{array}{c} 2\times \text{f} \end{array}}$$

Where,

V = Total volume of extract (5 ml)

f = Grams of fresh leaf (0.5g)

2 = Volume of extract taken (2 ml)

OD = Optical density measured as absorption reading

### Wax content

It was determined by the spectro-photometric method [29]. Ten leaf discs (2.5 cm^2^) from the top and middle leaf were immersed in 15 ml of chloroform for 15 seconds. The extract was filtered and evaporated to dryness on a boiling water bath, until the chloroform smell was completely vanished. Five ml of acidic potassium dichromate (K_2_Cr_2_O_7_) was added to the samples placed in boiling water bath for 30 minutes. After cooling to room temperature, 12 ml of deionized water was added and samples were left for 15 minutes and allowed for the colour development and then the optical density of the sample was read at 590 nm in a spectro-photometer (Elico, BL 222, Double beam). Wax was quantified by using standard curve obtained from carbowax 3000 and expressed in mgcm^-2^.

### Statistical analysis

The data recorded on various parameters during field experimentation was analyzed using the standard statistical procedures. The analysis of variance for split plot design was carried out as per the model proposed by [30] using R Studio agricolae (Version 4.2.1) statistical package.

$${\text{Y}}_{\text{ijk}}=\mu +{\;\text{p}}_{\text{i}}+\alpha_{\text{j}}+\delta_{\text{ij}}+\beta_{\text{k}}+{\left(\alpha \beta \right)}_{\text{jk}}+\varepsilon_{\text{ijk}}$$

where,

i = 1, 2,……..r

j = 1, 2, ……p

k = 1, 2, ……q

μ = General mean

p_i_ = Effect of i^th^ replication

α_j_ = Effect of j^th^ main plot treatment

δ_ij_ = Main plot error

β_k_ = Effect of k^th^ subplot treatment

(αβ)_jk_ = Interaction between j^th^main plot treatment and k^th^ subplot treatment

ε_ijk_ = Random error term occurring in split plot

Mean was calculated as the average of a set of numerical values. Mean value of each trait was worked out by dividing the sum total by the corresponding number of observations. Range gives the upper and lower limit of variability. It is measured as the difference between the highest and lowest mean value for each trait. Both phenotypic and genotypic coefficient of variability (PCV and GCV) for all the traits was estimated using the formulae of [31]. Heritability (broad sense) was estimated for all the traits as the ratio of genotypic variance to the total variance as suggested [32, 33]. Genetic advance as per cent of mean (GAM) was computed as the ratio of GA to the grand mean of the trait and expressed in percentage.

**Drought tolerance index (DTI)**: DTI was calculated by using the following formula as given by [34]

$$\text{DTI}=\frac{\left(\text{Ys}\;\text{x}\;\text{Yp}\right)}{{\left(\text{Yp}\right)}^{2}}$$

Where,

Ys—Mean yield of each inbred under water stress condition

Yp—Mean yield of each inbred under well watered condition

${\bar{\text{Y}}}_{\text{p}}$—Mean yield across the inbreds under well watered condition

The inbreds were classified as tolerant, moderately tolerant and susceptible based on drought tolerance index as shown under.


DroughtToleranceIndexToleranceCategory>0.9Tolerant0.8−0.9Moderate<0.8Susceptible

## Results

### Genetic variability and heritability

Analysis of variance revealed that mean sum of squares (MSS) due to water treatment and genotypes was significant for all the traits *viz*., relative water content (60 and 75 DAS), specific leaf weight (60 and 75 DAS), SPAD (60 and 75 DAS), proline content, wax content, pollen fertility, days to 50% tasselling, days to 50% silking, anthesis-silking interval, plant height, ear height, ear length, ear diameter, kernel row number, number of kernels per row, shelling percentage, harvest index, 100 grain weight and grain yield (Table 3). Besides, the MSS due to interaction between moisture treatment and genotype was also significant for all the traits except 100 grain weight (Table 3).

**Table 3:** Mean sum of squares due to different sources of variation for various traits of corn inbreds under water treatment.

Trait	Replication	Moisture α_j_	Error δ_ij_	Genotypes β_k_	Error ε_ijk_	Moisture × Genotype	Total
**Degrees of freedom**	1	1	1	27	54	27	111
**RWC 60 DAS**	0.186	2555.045**	2.185	589.767**	2.828	92.114**	190.278
**RWC 75 DAS**	10.165	1701.175**	1.344	537.047**	2.927	103.191**	172.587
**SLW 60 DAS**	0 x 10^−3^	8 x 10^−3^**	1 x 10^−3^	11 x 10^−3^**	0 x 10^−3^	2 x 10^−3^**	3.6 x 10^−3^
**SLW 75 DAS**	1 x 10^−3^	7 x 10^−3^**	0 x 10^−3^	5 x 10^−3^**	0 x 10^−3^	2 x 10^−3^**	2 x 10^−3^
**SCMR at 60 DAS**	3.832	776.103**	0.072	52.028**	0.856	18.952**	24.709
**SCMR at 75 DAS**	28.785	683.240**	3.967	34.395**	3.520	28.881**	23.554
**Proline**	0.451	733.489**	0.124	1.601**	0.050	1.373**	7.361
**Wax**	0.019	34.621**	0.009	0.256**	0.011	0.338**	0.462
**Pollen fertility**	1.150	94.172*	1.785	25.309**	0.128	2.756**	7.764
**PH**	54.781	14467.090**	0.187	1683.409**	17.251	265.088**	613.181
**EH**	3.526	4785.512**	0.961	420.216**	1.983	85.263**	167.072
**DFT**	16.509	130.723**	7.509	155.268**	1.750	12.760**	43.117
**DFS**	12.223	535.938**	3.938	144.490**	1.821	13.382**	44.261
**ASI**	0.321	137.286**	0.571	4.110**	0.798	1.619*	3.026
**Ear length**	0.008	70.814**	0.329	11.289**	0.198	1.028**	3.733
**Ear diameter**	0.232	7.016**	0.027	0.383**	0.033	0.222**	0.229
**KRN**	1.128	168.797**	0.012	7.555**	0.284	3.376**	4.328
**NKR**	0.941	581.017**	0.369	42.819**	2.224	16.343**	20.719
**Shelling Percentage**	0.212	344.329**	0.006	48.286**	0.163	10.413**	17.461
**100 grain weight**	0.438	214.509**	2.009	18.898**	1.760	2.861	8.103
**HI**	11.177	2391.719**	3.036	130.533**	7.769	24.681**	63.209
**GY**	0.012	43.525**	0	1.887**	0.116	0.493**	1.027

* and **—Significant at 0.05 and 0.01 level of probability, respectively.

RWC: Relative water content SLW: Specific leaf weight

SCMR–SPAD chlorophyll meter reading

PH: Plant height EH: Ear height

DFT—Days to 50% tasselling DFS: Days to 50% silking

ASI: Anthesis-silking interval KRN—Number of kernel rows per ear

NKR—Number of kernels per row HI: Harvest index GY—Grain yield

Phenotypic coefficient of variation (PCV) and genotypic coefficient of variation (GCV) were relatively higher under water stress condition over well watered condition for all the morpho- physiological, yield and yield components (Table 4). There was less difference between PCV and GCV for all the traits both under well watered (< 30%) and water stressed (< 35%) condition suggesting there was accuracy in the experimentation with minimal errors.

**Table 4:** Components of genetic variation for different traits under well watered and water stress condition of corn inbreds.

Sl. No.	Trait	PCV		GCV		H		GAM	
		WW	WS	WW	WS	WW	WS	WW	WS
1	RWC (%) at 60 DAS	13.4	22.6	13.1	22.2	99.4	97.9	27.4	45.2
2	RWC (%) at 75 DAS	14.3	23.5	13.9	22.1	94.7	97.6	27.9	48.6
3	SLW (g/dm^2^) at 60 DAS	14.1	14.4	13.3	13.2	90.5	83.5	26.2	24.8
4	SLW (g/dm^2^) at 75 DAS	9.2	9.9	8.3	9.2	81.8	85.68	15.5	17.6
5	SCMR at 60 DAS	7.5	12.2	7.2	11.8	95.4	95.2	14.7	23.8
6	SCMR at 75 DAS	8.6	12.1	7.9	10.6	84.4	77.1	14.9	19.3
7	Proline content (μ mol/g fr. Wt.)	8.4	13.4	7.1	13.0	71.3	95.1	12.3	26.2
8	Wax content (mg/cm^2^)	10.4	16.6	9.5	16.1	85.5	94.2	18.3	32.2
9	Pollen fertility (%)	2.2	3.3	2.1	2.8	96.7	98.8	4.4	6.8
10	Days to 50% tasseling	9.2	7.3	9.0	7.1	97.7	93.25	18.4	13.9
11	Days to 50% silking	8.5	6.6	8.3	6.4	97.5	92.51	17.1	12.6
12	Anthesis-Silking interval (days)	29.2	29.4	22.4	21.8	59.3	55.3	35.6	33.5
13	Plant height (cm)	17.5	19.1	17.1	18.8	95.5	97.8	34.3	38.4
14	Ear height cm)	19.8	15.3	19.7	15.1	98.9	97.3	40.3	30.6
15	Ear length (cm)	10.8	13.4	9.8	13.1	92.6	94.9	21.5	26.2
16	Ear diameter (cm)	10.6	11.6	10.0	9.9	85.64	73.9	21.8	17.7
17	No. of kernel rows	11.2	12.3	10.2	12.1	86.0	95.8	21.4	24.3
18	No. of kernels	13.3	19.1	13.0	17.8	83.7	87.6	27.4	34.4
19	Shelling per centage (%)	4.1	8.8	3.9	7.8	98.4	99.3	8.9	17.9
20	Hundred grain weight (g)	8.1	8.7	6.7	7.5	69.8	74.5	11.6	13.4
21	Harvest index (%)	9.8	23.2	8.7	21.5	71.0	86.3	17.1	41.1
22	Grain yield (tha^-1^)	28.8	46.3	25.4	43.2	78.0	87.3	46.3	83.2

DAS—Days after sowing WW- Well watered WS—Water stress RWC—Relative water content

SLW—Specific leaf weight SCMR: SPAD chlorophyll meter reading PCV—Phenotypic Coefficient of Variation

GCV—Genotypic Coefficient of Variation H—Heritability in broad sense GAM: Genetic advance over mean

All the traits under study except ASI showed high heritability both under well watered and water stress condition. Heritability estimates for RWC at 75 DAS, SLW at 75 DAS, proline content, wax content, pollen fertility, plant height, ear length, number of kernel rows, number of kernels per row, shelling percentage, hundred seed weight, harvest index and grain yield, increased under water stress compared to well watered condition. Higher genetic advance as percent of mean (>20%) was observed for RWC (both at 60 and 75 DAS), SLW at 60 DAS, anthesis silking interval, plant height, ear height, ear length, ear diameter, number of kernel rows, number of kernels per row and grain yield under both well watered and water stress conditions (Table 4).

### Response of corn inbreds to water treatments

#### Physiological traits

*Relative water content*, *specific leaf weight and SCMR*. Mean RWC was higher at 60 DAS both under both well watered and water stress condition compared to 75 DAS (Tables 5–7). Inbred IMIC 2030 showed very less reduction (0.9) under water stressed over well watered condition at 60 DAS while at 75 DAS, inbred PDM 4251 has showed 8.4 per cent increased RWC under water stress over well watered condition (Tables 5–7). No much difference in mean specific leaf weight (SLW) was observed between water treatments across inbreds. However, SLW among the different corn inbreds varied both under well-watered and water stress condition (Tables 5–7). The inbreds CML 563, CAL 1426–2 and PDM 4641 showed minimum reduction (6.2 to 10.6%) in specific leaf weight under water stress over well watered condition both at 60 and 75 DAS (Tables 5–7). The mean SPAD chlorophyll meter reading (SCMR) across inbreds was lower in water stress condition compared to well watered condition both at 60 and 75 DAS. However, inbred CML 563 had higher SCMR under water stress compared to well watered condition both at 60 and 75 DAS. Inbreds, CML 579–2, PDM 77–4 and PDM 4251 showed less reduction in SCMR both at 60 and 75 DAS (Tables 5–7) over well watered condition.

**Table 5:** Performance of Corn inbreds and per cent reduction over well watered for physiological and biochemical traits.

Inbred	Relative water content (%)						Specific leaf weight (g/dm^2^)						SPAD						Pollen fertility (%)			Proline content (μ mole/g fr. wt.)			Wax content (mg/cm^2^)		
	60 DAS			75 DAS			60 DAS			75 DAS			60 DAS			75 DAS											
	WW	WS	% C	WW	WS	% C	WW	WS	% C	WW	WS	%R	WW	WS	% C	WW	WS	% C	WW	WS	% C	WW	WS	% I	WW	WS	% I
**PDM 77–4**	67.7^m^	43.8^m^	-35.3	60.5^jk^	38.8^p^	-38.3	0.36^fg^	0.35^i^	-2.7	0.45^c-j^	0.43^gh^	-4.4	43.4^ijk^	42.8^efg^	-1.4	36.5^j^	36.6^fgh^	0.3	90.8^k^	89.9^lm^	-0.9	4.00^a-f^	6.44^l^	61.0	1.94^f-l^	2.11^l^	8.7
**PDM 260–1**	77.6^ij^	69.2^fg^	-10.8	63.5^hijk^	57.7^i^	-11.4	0.33^g^	0.34i	3.0	0.46^c-g^	0.44^fgh^	-5.1	41.1^l^	39.8^h-k^	-3.1	39.2^ij^	36.3f^gh^	-7.3	90.5^k^	87.5^o^	-3.3	4.07^a-f^	6.88^kl^	69.1	1.92^f-l^	2.99^fgh^	55.7
**PDM 4341**	80.6^g^	67.9^e^	-15.7	78.9^cd^	69.7^f^	-11.6	0.42^cde^	0.45^d-g^	7.2	0.46^c-g^	0.48^cde^	4.9	43.5^ijk^	41.4^fghi^	-4.8	42.1^d-i^	39.9^b-g^	-5.1	92.8^j^	89.7^m^	-3.3	4.15^a-d^	7.18^k^	73.0	1.81^kl^	2.82^hi^	55.8
**PDM 4251**	76.3^j^	70.1^fg^	-8.2	64.3^hijk^	69.7^f^	8.4	0.43^bcd^	0.44^d-g^	2.3	0.45^c-i^	0.46^d-h^	1.3	48.2^c^	46.5^bc^	-3.4	44.1^c-g^	43.7^ab^	-0.8	94.4^h^	90.1^lm^	-4.6	3.99^a-f^	7.98^hij^	100.0	1.75^l^	3.13^defg^	78.8
**PDM 4641**	94.5^a^	90.6^a^	-4.2	87.7^a^	83.9^b^	-5.5	0.55^a^	0.51^bc^	-7.3	0.57^a^	0.53^b^	-6.2	48.2^c^	42.1^e-h^	-12.6	43.7^c-h^	41.3^a-f^	-5.6	98.6^a^	97.9^a^	-0.7	3.91^a-g^	11.27^a^	188.2	1.91^f-l^	3.84^a^	101.0
**PML 17**	84.8^ef^	63.4^hi^	-25.2	75.6^c-f^	78.4^d^	0.4	0.38^ef^	0.43^d-h^	13.2	0.43^e-j^	0.43^gh^	1.2	45.7^fgh^	39.4^ijk^	-13.8	42.7^c-i^	37.3^fgh^	-12.8	96.9^de^	95.5^fgh^	-1.4	3.71^e-h^	9.93^c^	167.6	2.22^bcd^	3.32^cde^	49.5
**PML 46**	83.5^f^	71.9^f^	-13.8	75.9^c-f^	67.0^g^	-13.7	0.36^fg^	0.39^ghi^	8.3	0.42^g-k^	0.42^h^	-1.2	44.1^hij^	40.7^g-j^	-7.5	41.1^f-i^	36.5^fgh^	-11.2	95.9^fg^	94.8^hij^	-1.1	3.81^c-h^	8.81^fg^	131.2	2.20^cde^	3.16^def^	43.6
**PML 93**	80.7^g^	63.6^hi^	-21.2	77.3^cde^	58.4^i^	-26.9	0.44^bc^	0.46^c-f^	4.5	0.46^c-h^	0.51^bcd^	9.9	42.6^jkl^	39.9^h-k^	-6.3	40.6^ghi^	37.6^d-h^	-7.5	96.3^ef^	94.3^j^	-1.9	3.22^i^	7.89^hij^	145.0	2.44^a^	3.05^efgh^	25.0
**PML 54**	76.0^p^	69.9^fg^	-3.6	51.8^l^	47.9^m^	-14.2	0.46^bc^	0.62^a^	34.8	0.48^bcd^	0.62^a^	27.8	42.2^kl^	37.81^k^	-10.4	41.7^e-i^	40.4^b-g^	-3.1	96.2^efg^	95.7^efg^	-0.5	3.7^fgh^	8.84^fg^	138.9	1.88^g-l^	2.83^hi^	50.5
**PML 102**	90.5^d^	58.2^jk^	-2.5	66.6^hi^	45.3^n^	-35.8	0.45^bc^	0.47^cde^	4.4	0.49^bc^	0.45^e-h^	-9.1	48.1^cd^	44.3^cde^	-7.68	42.4^d-i^	42.9^a-d^	1.2	93.7^hi^	91.2^k^	-2.7	3.56^ghi^	9.79^cd^	175.0	2.00^fghij^	2.40^jkl^	20.0
**DIM 204**	61.8^n^	87.7^gh^	41.9	61.9^ijk^	62.8^h^	0.6	0.36^fg^	0.4^f-i^	11.1	0.45^cd-j^	0.44^gh^	-3.3	46.2^efg^	32.3^m^	-30.0	44.6^cde^	32.5^hi^	-27.2	96.8^de^	95.5^fgh^	-1.4	4.04^a-f^	9.63^cd^	138.3	2.04^e-h^	2.79^hi^	36.7
**DIM 102**	70.3^l^	43.5^m^	-38.1	67.3^gh^	42.1^o^	-38.2	0.42^cde^	0.4^f-i^	-4.7	0.46^c-g^	0.42^h^	-10.7	44.3^hij^	40.9^ghi^	-7.5	42.3^d-i^	38.2^c-g^	-9.6	97.2^cd^	96.4^cde^	-0.7	3.47^hi^	8.83^fg^	154.4	2.23^bc^	2.86^ghi^	28.25
**CDM 112**	59.6^o^	78.7^e^	32.1	50.7^l^	49.5^l^	-7.4	0.45^bc^	0.43^d-h^	-4.4	0.46^c-g^	0.45^e-h^	-2.2	44.9^ghi^	34.7^l^	-22.4	42.3^d-i^	32.6^hi^	-22.7	97.2^cd^	96.4^cde^	-0.8	4.11^a-f^	8.65^fg^	110.4	1.83^jkl^	3.78^ab^	106.5
**D 2287**	66.5^m^	53.1^l^	-20.2	59.5^k^	48.7^lm^	-20.5	0.37^fg^	0.39^ghi^	5.4	0.37^k^	0.42^h^	12.0	42.1^kl^	34.6^l^	-17.6	40.9^ghi^	32.7^hi^	-20.1	95.6^fg^	94.9^g-j^	-0.7	3.99^a-f^	8.92^efg^	123.5	2.38^ab^	3.72^ab^	56.3
**D 1013**	69.7^l^	57.6^jkl^	-17.4	63.3^h-k^	51.1^k^	-21.6	0.42^cde^	0.44^d-g^	4.7	0.47^cdef^	0.47^d-g^	0	50.9^b^	46.7^bc^	-8.3	49.9^a^	32.9^hi^	-34.2	95.7^fg^	94.3^j^	-1.5	4.13^a-e^	9.2^def^	122.7	1.95^f-k^	3.33^cde^	70.7
**CM 111**	79.9^gh^	78.3^m^	-1.6	77.7^cd^	43.1^o^	-45.3	0.45bc	0.49^bcd^	8.8	0.43^e-j^	0.52^bc^	22.1	51.9^b^	42.5^efg^	-18.2	48.4^ab^	37.7^d-h^	-22.1	97.5^bcd^	96.6^c^	-0.9	4.3^a^	9.81^cd^	128.1	1.86^h-l^	3.51^bc^	88.7
**GPM 114**	91.3^b^	88.0^abc^	-3.6	84.4^ab^	81.8^c^	-3.3	0.43^bcd^	0.42^e-h^	-2.3	0.42^h-k^	0.46^d-h^	10.8	50.6^b^	45.5^cd^	-10.1	50.3^a^	42.7^a-e^	-15.1	97.7^bcd^	97.4^ab^	-0.2	3.79c^-h^	10.52^b^	177.5	1.75^l^	3.90^a^	122.8
**CML 451**	78.7^hi^	65.9^gh^	-16.3	71.6^fg^	66.3^g^	-7.5	0.56^a^	0.53^b^	-5.3	0.52^b^	0.49^b-e^	-5.7	46.1^efg^	43.7^def^	-4.9	39.5^ij^	40.1^b-g^	1.2	95.3^g^	88.6^n^	-7.1	4.2^abc^	10.46^b^	157.8	1.89^f-l^	2.66^ij^	40.7
**CAL 1426–2**	91.7^b^	89.8^ab^	-2.1	85.0^ab^	85.9^a^	1.1	0.47^b^	0.43^d-h^	-8.5	0.47^cde^	0.44^fgh^	-7.4	48.4^c^	44.1^de^	-8.9	41.1^ghi^	45.8^a^	11.6	97.6^b-d^	95.8^def^	-1.8	4.13^a-e^	10.83^ab^	129.0	1.85^i-l^	3.77^ab^	103.7
**CML 563**	73.9^k^	69.9^ij^	-5.4	63.2^h-k^	54.9^j^	-15.5	0.47^b^	0.42^e-h^	-10.6	0.47^cdef^	0.49^b-e^	5.3	46.5^def^	48.1^ab^	3.4	43.5^c-h^	44.7^ab^	2.7	95.4^fg^	90.6^kl^	-4.9	4.04^a-f^	9.24^def^	128.7	2.03^e-i^	2.65^ij^	30.5
**IMIC 2030**	91.1^b^	90.2^a^	-0.9	84.9^ab^	80.8^c^	-6.01	0.36^fg^	0.46^c-f^	27.7	0.41^ijk^	0.48^c-f^	18.3	44.3^hij^	41.8^e-i^	-5.5	40.8^ghi^	39.9^b-g^	-2.1	97.3^cd^	96.5^cd^	-0.7	3.87^b-h^	7.86^ij^	103.1	2.24^bc^	2.31^kl^	3.12
**CML 579–1**	89.2^c^	82.2^de^	-7.8	76.6^cde^	78.8^d^	-1.7	0.38^ef^	0.44^d-g^	15.7	0.42^fghij^	0.52^bc^	22.4	44.3^hij^	42.7^efg^	-3.3	40.2^hi^	40.7^a-f^	1.2	95.4^fg^	94.4^ij^	-1.1	3.69^fgh^	8.63^fg^	133.8	2.38^ab^	2.60^ij^	9.24
**CML 579–2**	71.0^l^	70.1^fg^	-1.4	62.2^ijk^	64.1^h^	3.1	0.42^cde^	0.44^d-g^	4.7	0.44^d-j^	0.49^cde^	10.1	43.7^ijk^	43.2^d-g^	-1.1	41.4^e-i^	39.5^b-g^	-4.7	95.5^fg^	95.2^fghi^	-0.4	3.29^i^	8.43^ghi^	156.2	1.87^g-l^	2.47^jk^	32.0
**CML 580**	77.8^ij^	75.9^e^	-2.4	72.6^ef^	72.6^e^	-2.1	0.39^def^	0.4^f-i^	2.5	0.42^g-k^	0.45^e-h^	7.2	55.1^a^	49.3^a^	-10.3	51.5^a^	43.5^abc^	-15.7	95.6^fg^	90.6^kl^	-5.2	3.22^i^	7.83^j^	143.1	1.79^kl^	3.20^def^	78.7
**CML 582**	86.7^d^	81.2^de^	-6.3	75.5^def^	71.1^f^	-9.9	0.35^fg^	0.37^hi^	5.7	0.43^e-j^	0.42^gh^	-2.3	47.5^cde^	38.2^jk^	-19.5	46.1^bc^	37.4^e-h^	-18.8	93.1^ij^	89.9^lm^	-3.3	3.72^e-h^	8.46^gh^	127.4	2.05^d-g^	3.63^ab^	77.0
**IMIC 2024**	91.6^b^	85.1^bcd^	-7.1	80.6^bc^	81.2^c^	3.8	0.35fg	0.34^i^	-2.8	0.41^jk^	0.43^gh^	7.4	47.6^cde^	38.3^jk^	-19.5	44.6^c-f^	35.3^gh^	-20.9	98.3^ab^	96.9^bc^	-1.3	3.76^d-h^	10.69^b^	184.3	1.87^g-l^	3.70^ab^	97.8
**PML 9**	69.9^l^	61.6^m^	-40.5	65.1^hij^	39.4^p^	-44.1	0.38^ef^	0.41^e-h^	7.8	0.42^h-k^	0.48^c-f^	16.8	41.3^l^	30.8^m^	-25.3	39.7^ij^	30.1^i^	-24.4	97.9^abc^	97.5^ab^	-0.4	4.24^ab^	9.75^cd^	129.9	1.86^h-l^	3.35^cd^	80.1
**PML 21**	85.4^de^	79.7^e^	-6.7	75.2^def^	70.6^f^	-6.1	0.43^bcd^	0.41^e-h^	-2.7	0.49^bc^	0.46^d-h^	-7.1	48.1^cd^	30.5^m^	-1.5	45.3^cd^	29.7^i^	0.30	97.3^cd^	96.8^bc^	-0.9	3.92^a-e^	9.6^cd^	144.8	2.07^c-f^	3.27^c-f^	57.9
**Mean**	**78.0**	**68.5**		**70.7**	**62.9**		**0.41**	**0.42**		**0.45**	**0.47**		**46.09**	**40.8**		**43.1**	**38.2**	**46.1**	**95.8**	**93.9**		**3.85**	**9.01**		**1.99**	**3.11**	
**CD (5%)**	**0.70**	**4.90**		**4.90**	**1.40**		**0.04**	**0.05**		**0.04**	**0.04**		**1.5**	**2.21**		**3.00**	**4.50**	**1.5**	**0.77**	**0.71**		**0.35**	**0.54**		**0.16**	**0.25**	
**CV (%)**	**1.10**	**3.25**		**3.30**	**1.10**		**4.40**	**5.90**		**3.90**	**3.80**		**1.59**	**2.64**		**3.4**	**5.8**	**1.6**	**0.39**	**0.37**		**4.48**	**2.94**		**3.94**	**3.97**	

DAS—Days after sowing WW—Well watered condition WS—Limited Water stress condition % C—Per cent Change over control I—Increase % I—Per cent increase

Note: The genotypes with same superscripts do not differ signficantly at 5 per cent level of probability which is based on Duncan’s Multiple Range Test (DMRT)

**Table 6:** Performance of corn inbreds and per cent reduction over well watered for phenological traits.

Inbred	Days to 50% tasseling (days)			Days to 50% silking (days)			ASI (days)			Plant height (cm)			Ear height (cm)		
	WW	WS	I (days)	WW	WS	I (days)	WW	WS	I (days)	WW	WS	% C	WW	WS	% C
**PDM 77–4**	76.5^ghi^	86.0^bcde^	10.5	82.0^ghij^	92.5^abc^	5.5	5.5^a^	6.5^d^	1.0	101.4^kl^	85.7^jk^	-15.5	46.8^m^	38.1^m^	-18.5
**PDM 260–1**	72.0^j^	75.5^ijk^	6.5	76.5^nop^	83.0^gh^	4.5	4.5^b^	7.5^b^	3.0	100.1^l^	102.9^gh^	2.8	43.3^n^	46.2^j^	6.7
**PDM 4341**	79.5^f^	86.0^bcde^	9.0	84.0^gh^	93.0^abc^	4.5	4.5^b^	7^c^	2.5	125.9^hi^	98.8^hi^	-21.5	65.2^j^	42.9^kl^	-34.2
**PDM 4251**	79.0^f^	79.0^ghi^	2.0	83.5^gh^	85.5^efg^	4.5	4.5^b^	6.5^d^	2.0	135.0^efgh^	113.9^f^	-15.6	70.1^i^	55.6^fg^	-20.6
**PDM 4641**	72.0^j^	74.0^jk^	2.0	74.5^pq^	76.5^k^	2.5	2.5^f^	2.5^j^	0.0	99.5^l^	95.2^i^	-4.3	41.7^n^	53.2^gh^	27.5
**PML 17**	76.0^ghi^	81.0^g^	6.0	79.5^ijklm^	85.5^efg^	3.5	3.5^d^	4.5^g^	1.0	98.1^l^	77.4^l^	-21.2	54.2^l^	47.2^j^	-12.8
**PML 46**	75.5^i^	75.5^ijk^	2.0	79.0^lmn^	81.0^hij^	3.5	3.5^d^	5.5^e^	2.0	118.7^ij^	95.6^i^	-19.4	62.2^k^	47.7^j^	-23.2
**PML 93**	78.5^fgh^	79.0^ghi^	2.5	82.0^ghi^	84.5^fgh^	3.5	3.5^d^	5.5^e^	2.0	113.1^j^	92.3^ij^	-18.3	56.2^l^	45.4^jk^	-19.1
**PML 54**	78.5^fg^	75.5^ijk^	-0.5	81.5^hijk^	81.0^hij^	3	3^e^	5.5^e^	2.5	133.8^fgh^	127.2^cd^	-4.9	78.5^def^	66.3^b^	-15.4
**PML 102**	81.0^ef^	79.0^ghi^	-2.0	84.5^fg^	82.5^fgh^	3.5	3.5^d^	3.5^i^	0.0	134.0^fgh^	97.9^hi^	-26.9	73.4^gh^	59.3^de^	-19.2
**DIM 204**	74.0^ij^	78.0^hij^	5.5	77.0^mno^	82.5^gh^	3.0	3^e^	4.5^g^	1.5	111.9^jk^	83.8^k^	-25.1	56.5^l^	48.3^ij^	-14.5
**DIM 102**	65.5^l^	72.0^k^	8.5	69.0^s^	77.5^jk^	3.5	3.5^d^	5.5^e^	2.0	109.5^jkl^	102.6^gh^	-6.3	56.3^l^	52.1^h^	-7.5
**CDM 112**	69.5^k^	75.5^ijk^	9.5	72.5^qr^	82.0^ghi^	3.0	3^e^	6.5^d^	3.5	118.7^ij^	76.0^l^	-35.9	54.9^l^	42.1^l^	-23.3
**D 2287**	72.5^j^	79.5^gh^	10.0	76.0^op^	86.0^efg^	3.5	3.5^d^	6.5^d^	3.0	131.2^gh^	87.4^jk^	-33.3	66.1^j^	51.6^h^	-21.9
**D 1013**	76.0^ghi^	76.5^hij^	2.5	78.5^lmno^	81.0^hij^	2.5	2.5^f^	4.5^g^	2.0	160.8^ab^	123.5^de^	-23.2	82.1^bc^	61.0^cd^	-25.7
**CM 111**	68.5^k^	74.5^jk^	6.0	72.0^r^	78.0^ijk^	3.5	3.5^d^	3.5^i^	0.0	142.8^def^	103.6^gh^	-27.4	73.4^gh^	57.5^ef^	-21.7
**GPM 114**	89.0^bc^	89.5^ab^	2.5	90.5^cd^	93.0^abc^	1.5	1.5^h^	4^h^	2.5	152.9^bcd^	114.0^f^	-25.4	83.3^b^	63.3^bc^	-23.9
**CML 451**	84.5^d^	85.5^e^	2.0	88.5^de^	90.5^bcd^	4.0	4^c^	5^f^	1.0	107.6^jkl^	132.9^c^	23.5	75.9^fg^	60.7^cd^	-20.1
**CAL 1426–2**	74.0^ij^	79.0^ghi^	3.5	77.5^mno^	81.0^hij^	3.5	3.5^d^	2^k^	-1.5	154.7^bc^	132.8^c^	-14.1	73.6^gh^	65.9^b^	-10.5
**CML 563**	75.5^i^	81.5^g^	11.0	79.5^ijklm^	90.5^bcd^	4.0	4^c^	9^a^	5.0	163.8^ab^	134.3^c^	-18.1	91.9^a^	64.4^b^	-29.9
**IMIC 2030**	78.5^fgh^	77.5^hij^	1.5	80.5^ijkl^	82.0^ghi^	2.0	2^g^	4.5^g^	2.5	160.1^ab^	104.6^gh^	-34.6	79.3^cde^	51.0^hi^	-35.6
**CML 579–1**	88.0^c^	89.0^abcd^	3.0	91.5^c^	94.5^ab^	3.5	3.5^d^	5.5^e^	2.0	170.3^a^	141.7^b^	-16.7	72.2^hi^	59.1^de^	-18.2
**CML 579–2**	91.0^b^	90.0^a^	0.5	94.5^a^	95.0^a^	3.5	3.5^d^	5^f^	1.5	164.2^ab^	142.1^b^	-13.4	89.7^a^	58.5^def^	-34.8
**CML 580**	90.5^b^	90.0^a^	-0.5	92.5^bc^	92.0^abc^	2.0	2^g^	2^k^	0.0	145.8^cde^	132.1^c^	-9.4	71.0^hi^	56.3^ef^	-20.7
**CML 582**	89.5^a^	94.0^abc^	2.0	94.0^ab^	96.0^ab^	4.5	4.5^b^	2^k^	-2.5	141.8^defg^	132.4^c^	-6.6	77.2^ef^	59.1^de^	-23.5
**IMIC 2024**	83.0^de^	85.0^ef^	3.0	86.5^ef^	89.5^cde^	3.5	3.5^d^	4.5^g^	1.0	167.9^a^	149.5^a^	-10.9	81.2^bcd^	64.7^b^	-20.3
**PML 9**	79.5^f^	82.0^fg^	4.0	83.5^gh^	87.5^def^	4.0	4^c^	5.5^e^	1.5	130.5^gh^	107.1^g^	-17.9	59.6^k^	60.9^cd^	2.1
**PML 21**	74.0^ij^	74.0^jk^	0.5	77.5^mno^	78.0^ijk^	3.5	3.5^d^	4.0^h^	0.5	146.5^cd^	117.1^ef^	-15.5	79.9^cde^	71.2^a^	-18.5
**Mean**	**78.2**	**80.8**		**81.7**	**85.7**		**3.50**	**4.90**		**133.6**	**110.8**		**68.4**	**55.3**	
**CD (5%)**	**2.23**	**3.21**		**2.25**	**3.67**		**1.29**	**1.25**		**10.2**	**6.4**		**2.94**	**2.8**	
**CV (%)**	**1.38**	**1.95**		**1.34**	**2.07**		**18.6**	**19.6**		**3.7**	**2.8**		**2.09**	**2.5**	

DAS—Days after sowing WW—Well watered condition WS—Limited Water stress condition % C—Per cent Change over control I—Increase % I—Per cent increase

Note: The genotypes with same superscripts do not differ significantly at 5 per cent level of probability which is based on DMRT

**Table 7:** Performance of corn inbreds and per cent reduction over well watered for yield and yield components.

Inbred	Ear length (cm)			Ear diameter (cm)			No. of kernel rows			No. of kernels per row			Shelling percentage (%)			Harvest index (%)			100 grain weight (g)			Grain yield (tha^-1^)		
	WW	WS	% C	WW	WS	% C	WW	WS	% C	WW	WS	% C	WW	WS	% C	WW	WS	% C	WW	WS	% C	WW	WS	% C
**PDM 77–4**	13.9^ij^	14.5^d-g^	3.7	3.5^ijkl^	3.4^b-g^	-3.2	17.1^bc^	12.6^e-i^	-26.0	24.6^i-l^	23.2^efgh^	-5.6	80.7^l^	76.3^lm^	-5.5	40.8^f-j^	31.2^d^	-23.7	26.5^f^	27.0^cd^	1.8	4.6^jkl^	0.8^kl^	-81.8
**PDM 260–1**	14.8^f-i^	12.4^jk^	-16.2	4.6^ab^	2.9^g^	-35.7	18.8^a^	12.4^f-i^	-33.7	29.4^cg^	22.1^gh^	-24.8	82.4^hi^	80.3^ghi^	-2.5	42.4^c-i^	37.1^cd^	-12.7	28.0^f^	27.5^cd^	-1.7	6.5^def^	0.8^kl^	-87.1
**PDM 4341**	12.9^kl^	11.2^l^	-13.8	3.5^ijkl^	3.2^d-g^	-8.0	14.2^e-h^	9.9^l^	-30.3	24.0^ijkl^	14.7^j^	-38.5	84.4^ef^	69.4^o^	-17.7	31.5^l^	14.1^f^	-55.2	29.5^cdef^	28.0^bcd^	-5.0	4.8^jk^	1.5^jkl^	-69.6
**PDM 4251**	15.7^d-g^	15.4^cd^	-2.1	4.4^bcd^	3.7^bcd^	-15.6	14.5^e-h^	12.2^ij^	-15.8	21.9^l^	15.7^j^	-28.3	81.6^ijkl^	75.2^mn^	-7.8	41.1^e-j^	36.7^cd^	-10.6	32.0^bcd^	28.5^bcd^	-10.9	2.9^n^	1.5^ijkl^	-50.0
**PDM 4641**	14.5^hi^	13.9^gh^	-4.3	3.9^e-h^	3.5^b-f^	-11.4	13.2^hi^	12.4^hi^	-6.1	24.2^i-l^	26.6^cde^	9.9	88.8^b^	87.3^b^	-1.8	47.7^a-d^	36.1^cd^	-24.4	33.5^ab^	31.0^b^	-7.4	6.7^def^	6.0^ab^	-9.3
**PML 17**	11.6^m^	8.9^m^	-22.6	4.1^c-f^	3.5^b-f^	-15.6	16.4^cd^	12.6^e-i^	-23.2	24.6^i-l^	17.8^ij^	-27.6	91.1^a^	87.8^b^	-3.5	42.3^d-i^	37.1^cd^	-12.1	29.5^cdef^	27.5^cd^	-6.7	3.3^mn^	2.7^fghi^	-18.7
**PML 46**	15.7^d-g^	13.1^hij^	-17.1	3.9^e-i^	3.7^b-e^	-5.4	13.6^f-h^	12.8^d-i^	-5.8	26.0^g-k^	22.8^fgh^	-12.0	77.5^m^	74.3^n^	-4.2	40.2^g-k^	37.9^cd^	-5.5	29.0^def^	26.5^d^	-8.6	4.4^kl^	2.3^ghij^	-47.6
**PML 93**	15.7^d-g^	14.2^fg^	-9.8	3.5^i-l^	3.2^efg^	-8.8	14.5^e-h^	13.3^cde^	-8.3	31.1^b-d^	24.9^cdefg^	-19.9	81.2^kl^	78.5^jk^	-3.2	45.9^a-g^	33.8^cd^	-26.3	29.5^cdef^	28.0^bcd^	-5.1	6.0^efg^	3.1^fgh^	-48.3
**PML 54**	15.6^d-h^	14.4^efg^	-8.2	3.8^f-j^	4.3^a^	14.5	19.5^a^	15.5^a^	-20.7	27.4^e-i^	22.7^fgh^	-17.1	82.5^ghi^	81.1^fgh^	-1.8	45.6^a-g^	38.1^cd^	-16.6	31.5^bcde^	27.5^cd^	-12.6	6.9^d^	4.4^cdef^	-36.3
**PML 102**	14.7^ghi^	12.8^ij^	-13.4	4.2^b-e^	3.2^efg^	-24.2	18.2^ab^	12.2^ij^	-32.9	25.7^h-k^	23.6^efgh^	-8.2	84.1^ef^	81.9^def^	-2.5	49.9^a^	46.3^a^	-7.3	29.5^cdef^	27.5^cd^	-6.7	9.8^a^	5.4^abc^	-38.3
**DIM 204**	12.3^lm^	11.2^l^	-9.6	3.4^jkl^	2.9^g^	-14.1	14.0^f-h^	8.0^m^	-42.8	25.0^ijkl^	16.4^j^	-34.4	87.0^c^	85.4^c^	-1.8	43.5^a-h^	34.4^cd^	-20.9	29.5^cdef^	28.5^bcd^	-3.3	5.2^hijk^	4.6^bcd^	-12
**DIM 102**	12.4^lm^	11.4^l^	-8.2	3.1^l^	2.3^h^	-24.8	11.6^i^	10.0^l^	-13.7	24.4^i-l^	20.0^hi^	-18.0	81.2^kl^	80.1^hi^	-1.4	37.2^h-l^	21.2^e^	-43.1	28.5^ef^	21.0^e^	-26.3	6.5^def^	0.4^l^	-93.5
**CDM 112**	13.5^jk^	12.5^jk^	-7.1	3.2^kl^	3.6^b-e^	13.1	14.6^e-h^	11.3^k^	-22.6	23.7^jl^	24.1^defg^	1.7	86.6^c^	81.1^fgh^	-6.4	35.6^jk^l	20.7^ef^	-41.9	28.5^ef^	28.0^bcd^	-1.7	5.8^efgh^	3.5^defg^	-35.7
**D 2287**	16.1^bcde^	13.9^gh^	-13.1	3.6^h-k^	3.2^efg^	-10.6	16.8^bc^	13.5^cd^	-19.9	28.6^d-h^	21.8^gh^	-23.7	84.9^de^	83.2^d^	-2.2	46.9^a-f^	44.4^ab^	-5.4	33.0^ab^	30.0^bc^	-9.1	7.1^d^	2.9^efgh^	-58.8
**D 1013**	14.7^ghi^	13.6^ghi^	-7.3	3.9^e-h^	3.5^b-f^	-11.3	13.4^gh^	12.3^hij^	-8.6	23.3^kl^	24.3^defg^	4.3	83.4^fg^	79.4^ij^	-4.7	41.5^d-j^	33.8^cd^	-18.4	33.5^ab^	29.5^bcd^	-11.9	7.9^b^	1.9^bcd^	-76.3
**CM 111**	15.2^e-h^	14.1^gh^	-7.4	4.1^c-g^	3.7^bcd^	-7.7	13.6^f-h^	12.4^hi^	-8.8	30.6^cde^	18.2^ij^	-40.5	76.2^n^	58.1^q^	-23.8	42.1^d-i^	33.1^cd^	-21.4	34.0^ab^	30.0^bc^	-11.7	7.7^bc^	5.2^abc^	-32.4
**GPM 114**	15.9^c-f^	15.2^cde^	-4.4	4.4^bc^	3.7^bc^	-14.7	15.0^defg^	11.6^jk^	-22.6	28.5^d-h^	17.6^ij^	-38.2	82.7^gh^	60.8^p^	-26.5	34.2^kl^	14.3^f^	-58	29.0^def^	26.5^d^	-8.6	7.1^ijk^	5.0^efg^	-14.3
**CML 451**	18.8^a^	16.4^b^	-12.9	4.1^c-g^	3.8^b^	-3.9	15.2^def^	14.4^b^	-5.3	32.4^bc^	23.8^defg^	-26.5	85.5^d^	81.5^efg^	-4.67	36.6^i-l^	36.6^cd^	-0.03	36.0^a^	35.0^a^	-2.7	5.2^ghij^	4.8^bcd^	-8.0
**CAL 1426–2**	18.4^a^	17.6^a^	-4.1	4.2^c-f^	3.8^b^	-6.9	15.0^d-g^	14.3^b^	-4.6	35.7^a^	32.4^a^	-9.3	87.4^c^	82.9^d^	-5.13	48.8^abc^	35.3^cd^	-27.7	33.5^ab^	28.5^bcd^	-14.9	8.5^b^	6.5^a^	-24.4
**CML 563**	16.7^bcd^	15.6^bc^	-6.7	4.8^a^	3.1^fg^	-35.6	15.8^cde^	13.0^defgh^	-17.7	34.1^ab^	28.3^bc^	-17.0	91.2^a^	89.3^a^	-2.05	47.0^a-f^	37.1^cd^	-21.2	32.0^bcd^	31.0^b^	-3.1	5.6^fghi^	3.5^cde^	-22.2
**IMIC 2030**	17.1^b^	12.9^ij^	-24.2	3.9^d-g^	3.5^b-f^	-12.0	14.8^e-h^	13.5^cd^	-8.7	31.1^bcd^	27.3^cd^	-12.2	82.2^hij^	80.4^ghi^	-2.27	42.9^b-i^	38.9^bc^	-9.2	32.0^bcd^	29.0^bcd^	-9.3	6.7^de^	3.8^defg^	-43.7
**CML 579–1**	16.7^bcd^	15.6^bc^	-7.1	4.1^c-f^	3.6^b-f^	-12.6	14.4^e-h^	12.4^hi^	-13.0	26.7^fghijk^	22.9^fgh^	-14.2	84.6^de^	82.7^de^	-2.26	42.1^d-i^	34.9^cd^	-16.8	32.5^bc^	27.5^cd^	-15.3	7.1^cd^	3.5^abc^	-44.1
**CML 579–2**	15.4^e-h^	13.6^ghi^	-11.7	4.1^c-g^	3.6^b-e^	-9.2	16.5^cd^	13.0^d-h^	-21.2	24.6^i-l^	26.2^cdef^	6.5	81.8^hijk^	78.4^jk^	-4.14	44.5^ab-g^	31.3^d^	-29.5	32.5^bc^	28.5^bcd^	-12.3	6.3^def^	3.3^bcd^	-46.6
**CML 580**	15.4^efgh^	15.1^c-f^	-2.1	4.2^bcde^	3.3^c-g^	-21.3	14.2^e-h^	13.0^d-h^	-8.4	27.2^e-j^	22.4^gh^	-17.6	85.1^de^	80.7^fghi^	-5.0	49.4^ab^	33.3^cd^	-32.4	33.5^ab^	29.5^bcd^	-11.9	4.8^jk^	3.5^defg^	-26.1
**CML 582**	16.9^bc^	14.3^efg^	-15.6	4.0^cdefg^	3.5^b-f^	-11.3	14.0^f-h^	14.0^bc^	0.0	29.8^c-f^	23.0^efgh^	-22.8	82.4^hi^	78.4^jk^	-4.9	42.6^c-i^	35.7^cd^	-16.1	33.0^ab^	29.0^bcd^	-12.1	4.0^lm^	3.3^efg^	-15.8
**IMIC 2024**	15.5^e-h^	11.8^kl^	-23.2	3.6^ghij^	3.3^c-g^	-7.9	14.5^e-h^	13.2^def^	-8.9	27.3^e-i^	22.0^gh^	-19.4	81.3^jkl^	77.3^kl^	-4.9	45.2^a-g^	34.5^cd^	-23.4	29.5^cdef^	26.5^d^	-10.2	4.8^jk^	3.3^efg^	-30.4
**PML 9**	14.7^gh^i	12.9^ij^	-12.5	4.2^bcdef^	3.6^b-f^	-14.5	15.0^d-g^	15.1^a^	0.6	28.5^defgh^	23.0^efgh^	-19.2	84.6^de^	81.3^fgh^	-5.5	42.9^b-i^	33.7^cd^	-21.3	32.5^bc^	30.0b^c^	-7.6	8.1^b^	4.0^defg^	-56.4
**PML 21**	15.3^e-h^	13.6^ghi^	3.7	3.9^efgh^	3.4^b-f^	-3.2	14.4^e-h^	13.2^d-g^	-26.0	25.6^hijk^	30.6^ab^	-5.6	84.2^ef^	75.9^m^	-2.5	47.6^a-e^	37.6^cd^	-23.7	34.0^ab^	31.0^b^	-8.8	8.1^b^	4.4^cdef^	-46.2
**Mean**	**15.2**	**13.6**		**3.9**	**3.43**		**15.1**	**12.6**		**27.3**	**22.8**		**83.8**	**78.9**	**42.8**	**42.8**	**33.5**		**31.3**	**28.5**		**6.2**	**3.4**	
**CD (5%)**	**0.96**	**0.85**		**0.00**	**0.41**		**1.4**	**0.65**		**2.9**	**3.2**		**0.89**	**1.21**	**5.52**	**5.52**	**5.9**		**2.8**	**1.5**		**0.8**	**0.3**	
**CV (%)**	**3.08**	**3.04**		**4.05**	**5.9**		**4.5**	**2.52**		**5.3**	**6.7**		**0.52**	**0.75**	**6.29**	**6.29**	**8.5**		**4.5**	**4.4**		**13.5**	**16.5**	

DAS—Days after sowing WW—Well watered condition WS—Limited Water stress condition % C—Per cent Change over control I—Increase % I—Per cent increase

Note: The genotypes with same superscripts do not differ significantly at 5 per cent level of probability which is based on DMRT

*Pollen fertility*, *proline and wax content*. In case of pollen fertility, no much difference could be observed between well watered and water stress condition. However, the inbreds, GPM 114, CML 579–2 and PML 54 recorded higher pollen fertility under well-watered condition with less reduction (<0.5% reduction) under water stress (Tables 5–7). In contrast to the above traits, the mean proline content of inbreds was higher (9.01 μ mole/g fresh weight) under water stress condition compared to well-watered condition (3.85 μ mole/g fresh weight). Also, all the inbreds, without any exception, accumulated higher proline content under water stress. The proline accumulation was highest under water stress condition in PDM 4641 (11.27 mole/g fresh weight) followed by IMIC 2024, GPM 114 and PML 102. These inbreds also recorded highest percentage increase over well watered condition in the same order. Wax content was higher under water stress condition over well watered condition across corn inbreds. The inbreds GPM 114, CDM 112, CAL 1426–2 and PDM 4641 accumulated more than double the wax content (Tables 5–7) under water stress condition compared to well-watered condition.

#### Phenological traits

The mean anthesis silking interval (ASI) was higher (4.9 days) under water stress condition as compared to well watered condition (3.5 days). Corn inbreds CML 582 and CAL 1426–2 had narrow ASI under water stress compared to well watered condition. The inbreds PDM 4641, PML 102, CM 111 and CML 580 recorded exactly the same ASI both under well watered and water stress condition. The plants, in general, were taller under well watered condition (133.6 cm) compared to water stressed condition (110.8 cm). CML 451 and PDM 260–1 have higher plant height under water stress condition compared to well watered condition (Tables 5–7). Corn inbreds PDM 4641, PML 54, DIM 102, CML 582 and CML 580 showed lower reduction in plant height (< 10%) under water stress condition (Tables 5–7). The mean ear height of all inbreds was higher under well watered condition (68.4 cm) compared to water stressed condition (55.3 cm). In contrast, there was increase in ear height in the corn inbreds, PDM 4641 (27.5%), PDM 260–1 (6.7%) and PML 9 (2.1%) under water stress condition (Tables 5–7). The reduction in ear height was less in case of inbreds, DIM 102 (7.5%) and CAL 1426–2 (10.5%) under water stress condition over well watered condition.

#### Productivity traits and yield

The ear length was lower (13.6 cm) under water stress condition as against 15.2 cm in case of well watered condition. However, there was increase in ear length under water stress condition in case of PML 21 and PDM 77–4 while lower reduction under water stress condition in ear length (<5.0%) was noted in case of CML 580, PDM 4251, CAL 1426–2, PDM 4641 and GPM 114. There was less difference in mean ear diameter of inbreds between well watered and water stressed condition (Table 4). There was less reduction (<5.0%) in ear diameter in case of PML 21, PDM 77–4 and CML 451 under water stress condition. Interestingly, there was increase in ear diameter in two inbreds PML 54 and CDM 112 under water stress condition. There was higher number of kernel rows in case of well watered condition (15.1) compared to water stress condition (12.6) across inbreds. There was very less reduction in number of kernel rows in case of inbreds PML 9, CML 582 and CAL 1426–2 (< 5.0%). In general, there was more number of kernels per row in case of well watered condition (27.3) when compared to water stressed condition (22.8) across all the corn inbreds studied in the experiment. In contrast, number of kernels per row increased under water stress condition in case of PDM 4641, CML 579–2, D 1013 and CDM 112. Shelling percentage was less (78.9%) under water stress condition when compared to well watered condition (83.8%) across the corn inbreds. Among the twenty eight inbreds studied, DIM 102, DIM 204, PML 54 and PDM 4641 have showed very less reduction in shelling percentage under water stress condition over well watered condition (Tables 5–7). The hundred seed weight was lower (28.5 g) under water stressed condition when compared to well watered condition (31.3 g) across the studied corn inbreds. Inbreds PDM 77–4, PDM 260–1, CDM 112, CML 451, DIM 204 and PDM 4341 showed very less reduction in hundred seed weight (< 5.0%) under water stress condition over well watered condition. The harvest index was higher (42.8%) under well watered condition over water stressed condition (33.5%) across corn inbreds. Among the inbreds, CML 451, D 2287, PML 46, PML 102 and IMIC 2030 have showed less reduction (< 10%) in harvest index under water stress condition.

The mean grain yield was higher under well watered condition (6.2 tha^-1^) over water stress condition (3.4 tha^-1^). Inbreds, CML 451, PDM 4641, DIM 204 and GPM 114 showed less than 15% reduction in grain yield under water stress over well watered condition. Highest yielding inbreds under well watered condition, *viz*., PML 102 (9.8 tha^-1^) and CAL 1426–2 (8.5 tha^-1^) recorded highest reduction of 38.3% and 24.4%, respectively under water stress condition.

There was variation among the studied corn inbreds for days to fifty per cent tasseling and silking which may cause bias in the study of effect of water stress on different traits. In this regard, analysis of covariance between days to fifty per cent silking with physiological, phenological and productivity traits under well watered condition (S1 Table) indicated independent nature of silking with RWC at 60 DAS, SLW at 75 DAS, SCMR at both 60 and 75 DAS, wax content, pollen fertility, ASI, ear height, ear length, ear diameter, number of kernel rows, shelling percentage and harvest index. Hence, effect of water stress on these traits has been discussed in detail in the following section.

## Discussion

At the experiment site, during rabi 2020–21, only 10 mm rainfall was received during water stress treatment period. As a result, the soil moisture got depleted from 26% at 15 DAS to 12.3% at 75 DAS in the water stressed plots while it remained around 25% under well-watered conditions. Thus, the sufficient soil moisture stress required to screen genotypes under drought stress was simulated in the plots (Fig 1). The temperature and relative humidity during the drought simulation period was around the optimum and hence, the experimental results were not much compounded with heat stress effects (Table 2).

### Genetic variation among inbreds for water treatments

There were significant differences for various physiological, biochemical, phenological, yield and yield components among main factor (water treatment) and sub factor (genotypes) indicating variability in the studied inbreds both under well watered and water stressed conditions. [35–37] also reported significant differences among moisture stress treatment and among the genotypes studied in corn. There was also differential response of inbreds to well watered and water stressed treatments for these traits as indicated by significant interaction between moisture treatment and genotypes (Table 3). Large genetic variation was evident in the studied corn inbreds especially under water stress condition for RWC (both at 60 and 75 DAS), SCMR, proline content, wax content, harvest index and grain yield (Table 4) as indicated by higher PCV and GCV in comparison to well watered condition for the corresponding traits. This suggested that water stress increased the genetic variability among inbred lines by discriminating between tolerant and susceptible inbreds and hence the pool of inbreds used in the study provide an opportunity to select desirable inbreds for these physiological, phenological traits and yield. Under well watered condition, higher PCV and GCV (>20%) were observed for anthesis-silking interval and grain yield (Table 4). While, it was moderate (10–20%) for RWC (both at 60 and 75 DAS), SLW at 60 DAS, plant height, ear height, ear diameter, number of kernel rows and number of kernels per row under well watered condition. In case of water stressed environment, higher PCV and GCV were observed for RWC (both at 60 and 75 DAS), proline content, wax content, harvest index and grain yield (Table 4). Earlier, higher PCV and GCV were noted for anthesis-silking interval and grain yield under both water stress and well watered conditions [36, 38, 39].

High heritability in broad sense (>60%) noted for all the traits except with moderate heritability (30–60%) for ASI (59.3 and 55.3%, under well-watered and water stress conditions, respectively) under both the water treatments indicated that differential response of genotypes have heritable component. It is worth noting that the heritability estimates increased when inbreds were exposed to water stress for RWC at 75 DAS, SLW at 75 DAS, proline content, wax content, pollen fertility, plant height, ear length, number of kernel rows, number of kernels per row, shelling percentage, hundred seed weight, harvest index and grain yield. This implied that the simulated water stress discriminated different inbreds based on their ability to tolerate the stress and hence there was increase in the genetic variability among the inbreds for these traits. Although higher genetic advance as per cent of mean for these traits was noted under well watered condition, the GAM was much higher under water stress conditions. This indicated more pronounced differential response of different inbreds under water stress (Table 4). Furthermore, these results indicated the scope for selection for these drought responsive traits in the studied corn inbreds. High heritability was noted in previous studies for days to 50% silking and tasselling, plant height, ear length, ear diameter, kernel number per row and grain yield [36, 39–41]. The high heritability coupled with high GAM for plant height, ear height, ear length, kernel row number and grain yield under water stress condition was also noted earlier by [13, 39, 41, 42].

### Effect of drought on physiological traits

In the present study, the simulated drought during pre-tasseling to grain formation stages led to the reduction in the overall mean values of all the physiological traits like, RWC, SLW and SCMR (Tables 5–7). The reduction in RWC was low (<2.5%) in case of CML 579–2, CAL 1426–2 and CML 580 both at 60 and 75 DAS under water stress (Tables 5–7). [43] also reported reduction in RWC with the increasing stress. However, some inbreds either showed increase in some parameter or showed minimum reduction in some parameters compared to other inbreds. For instance, the inbred CML 563 had higher SCMR under water stress compared to well watered condition both at 60 and 75 DAS implying its ability to synthesise more chlorophyll even under moisture stress condition. Inbreds, CML 579–2, PDM 77–4 and PDM 4251 showed less reduction in SCMR both at 60 and 75 DAS (Tables 5–7) over well watered condition which shows their ability to maintain chlorophyll content even under water stress condition. [44, 45] reported up to 60 per cent reduction in chlorophyll content when the drought stress was induced at flowering stage in corn. Inbred PDM 4251 has showed 8.4 per cent increased RWC under water stress over well watered condition at 75 DAS (Tables 5–7) which could be due to its differential reaction to moisture stress that varies from tissue and developmental stage specific response among genotypes [46].

The pollen fertility got reduced under water stress condition. The reduction was not much significant implying low seed set under water stress and drought susceptibility of genotypes is due to the reasons other than pollen fertility *per se*.

The overall mean value for proline content suggested that all the inbreds accumulated higher proline content (9.01 μ mole/g fresh weight) under water stress condition than that under well-watered condition (3.85 μ mole/g fresh weight) suggesting a definite role of proline under water stress situation. In our study, very high increase in proline (> 175% over well watered conditions) was observed in PDM 4641, CML 582, GPM 114 and PML 102. Proline acts as a metal chelator, an anti-oxidative defence molecule and a signalling molecule during any stress in addition acting as an excellent osmolyte [47]. Hence, under stress, there will be over production of proline, which in turn maintains cell turgor or osmotic balance. This prevents electrolyte leakage and brings concentrations of reactive oxygen species (ROS) within normal range. Thus proline accumulation stabilizes membranes and prevents oxidative burst in plants [48]. [19] also reported accumulation of proline content in corn genotypes under drought stress. The enhanced wax content in some inbreds avoids transpiration loss during water stress situation and thus making them to be drought tolerant. Previously, [49] also reported increased wax content in the corn inbreds and hybrids leading to less reduction in grain yield under drought condition.

### Effect of drought on phenological traits

Drought affects the tassel and silk emergence which in turn increases the anthesis silking interval. Among the twenty eight inbreds studied, four inbreds PDM 4641, PML 102, CM 111 and CML 580 recorded exactly the same ASI (2.5 days) both under well watered and water stress conditions showing their ability to tolerate the moisture stress. Shorter ASI can avoid barrenness leading to increased partitioning of assimilates to the developing ear. Conventional selection for grain yield along with secondary traits like ASI resulted in improved tolerance of maize to drought [50]. [51] observed delayed silking under drought over supplemental irrigation in corn. [52] while studying OPVs and hybrids of corn under managed drought conditions noted genetic gains in hybrids due to earliness and reduced ASI. Strong correlation was noted between grain yield and ASI in the corn populations [21] and in F_2_ population [53] under drought. Plant height was also affected by simulated drought which is evident by reduction in plant height across all inbreds. [54–56] also reported effect of water stress in reducing the plant height in corn. The reduction in plant height could be because of the acclimatization of the corn plants to escape moisture stress and plants could start to divert assimilates from stem and utilize them for shoot/canopy growth [57] in order to increase the absorption of light.

### Effect of drought on productivity traits and grain yield

Due to the effect of drought on various morpho-physiological parameters, there will be change in the source sink relationship. This will lead to reduction in the yield component traits (ear length, number of kernel rows, number of kernels per row, shelling percentage and hundred seed weight). The previous study by [58] indicated significant reduction in number of ears per plant, grains per ear, 100-grain weight, grain yield and harvest index. Further, reduction in number of kernels per row and 100 kernel weight under stress were noted by [59] and [60], respectively. The harvest index also got reduced under water stressed condition. It can also be noted that, there was differential response among the inbreds for all the yield components and some inbreds had higher performance under well watered and recorded lower reduction under water stress. For instance, the inbreds CAL 1426–2 and PDM 4641 recorded higher value under well watered and minimum reduction under water stress for majority of the component traits. This might be due to higher RWC, SLW and wax content and narrow ASI in CAL 1426–2 and higher SLW, proline and wax content with narrow ASI in PDM 4641.

Yield stability under drought conditions is an important aspect and reduced grain yield due to water stress is a common occurrence [61]. Reduction in grain yield of 25% in case of inbreds [19] and 37% in case of hybrids [62] was reported under drought stress in corn. In the present study the mean grain yield was higher under well watered condition (6.2 tha^-1^) over water stress condition (3.4 tha^-1^) and water stress reduced the grain yield up to 93.5% (DIM 102). However, some inbreds tolerated stress and exhibited minimum yield reduction under stress. For example, the inbreds CML 451 (8%), PDM 4641 (9.3%), DIM 204 (9.3%) and GPM 114 (12%) recorded lesser reduction in grain yield under water stress. The minimum reduction in grain yield could be due to higher RWC, SLW, proline, wax content and lower ASI in these inbreds. The inbred CAL 1426–2, though showed higher (24%) reduction in yield under water stress, its grain yield was highest (6.5 tha^-1^) among others under the stress condition. This was because of less reduction in RWC, SLW, ASI in case of CAL 1426–2 and hence, can be used as donor in drought tolerant hybrid development. The inbred CML 563 has less reduction in SCMR while, inbreds PDM 4641 and GPM 114 have increased proline and wax content.

**Fig 2:**
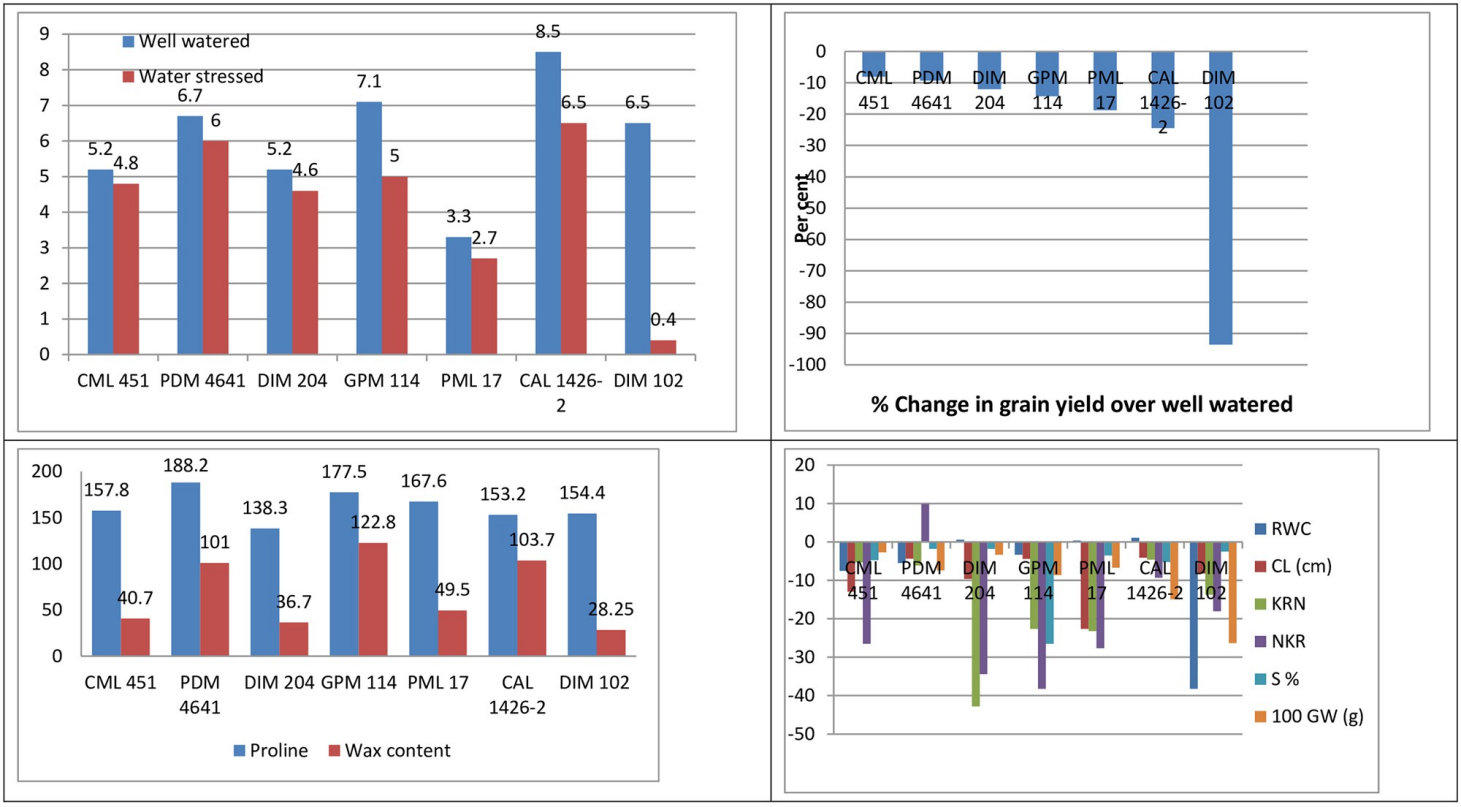
Per se performance of drought tolerant genotypes for grain yield (tha^-1^) and per cent change over well watered for yield, yield components and physiological parameters.

As far as productivity parameters are concerned, less reduction in ear length, number of kernel rows in case of CAL 1426–2, number of kernels per row and shelling percentage in case of PDM 4641, hundred seed weight, harvest index and grain yield was observed in case of CML 451. Different inbreds have different drought tolerant parameters and productivity parameters, which makes difficult to select the best drought tolerant inbred. Hence drought tolerance index (DTI) was considered to select drought tolerant inbred. Based on DTI, eight genotypes (PDM 4641, PML 102, CM 111, CAL 1426–2, PML 54, GPM 114, PML 9 and PML 21) were considered as drought tolerant/moderately tolerant (Table 8). Among these drought tolerant corn inbreds, CAL 1426–2 had higher *per se* performance for grain yield both under well watered (8.5 tha^-1^) and water stressed condition (6.5 tha^-1^; Fig 2). Inbreds GPM 114 and PDM 4641 have consistent *per se* performance in terms of grain yield both under well watered and water stress condition. These corn inbreds could be employed in hybrid development program suitable for rainfed cultivation.

**Table 8:** Grouping of corn inbreds based on drought tolerance index (DTI).

Range of DTI	Number of genotypes	Genotypes	Drought tolerance category
**> 0.9**	4	PDM 4641 (1.1), PML 102 (1.5), CM 111 (1.1), and CAL 1426 2 (1.5)	Tolerant
**0.8–0.9**	4	PML 54 (0.8), GPM 114 (0.9), PML 9 (0.9) and PML 21 (0.9)	Moderate
**< 0.8**	20	PDM 77-4(0.1), PDM 260-1(0.1), PDM 4341(0.2), PDM 4251(0.1), PML 17(0.2), PML 46(0.3), PML 93(0.5), DIM 204 (0.6), DIM 102(0.1), CDM 112(0.5), D 2287(0.5), D 1013(0.4), CML 451(0.7), CML 563(0.5), IMIC 2030(0.7), CML 579-1(0.7), CML 579-2(0.5), CML 580(0.4), CML 582(0.3) and IMIC 2024(0.4)	Susceptible

In summary, considering, physiological, phenological and productivity parameters, inbreds, CAL 1426–2 (higher RWC, SLW and wax, and lower ASI), GPM 114 (higher proline and wax) and PDM 4641 (higher SLW, proline and wax, and lower ASI) with different combination of drought tolerant mechanisms, higher/consistent productivity and higher drought tolerance index (>0.8) are potential drought tolerant inbreds in the present study. These inbreds may be used to produce experimental hybrids and to identify heterotic hybrids suitable for cultivation under rainfed ecosystem. Drought tolerant inbreds should be used as testers to know the inbreds potential in giving heterotic hybrids. These inbreds can be also used as testers to develop hybrids with other identified potential inbreds. Through, diallel mating among identified tolerant inbreds or line x tester mating design, more number of potential inbreds can be used to develop potentially water stress tolerant hybrids. Simultaneously, these inbreds could be intermated to combine different drought tolerant mechanisms and population improvement procedure may be followed to improve drought tolerance further. Identified drought tolerant inbreds in the study had higher grain yield which could be associated with existence of one or more drought tolerance parameters (proline content, wax content, ASI, RWC) in these inbreds. Hence, proline content, wax content, ASI, RWC can be used as better surrogate traits to identify drought tolerant inbreds in corn. The study also identified high yielding inbreds under well watered conditions. For example, PML 102, CAL 1426–2, PML 21, PML 9, D 1013 and CM 111. These could be used as female parents in heterosis breeding programmes of corn to develop corn hybrids specifically suited to irrigated ecologies.

## Conclusion

Twenty eight corn inbreds studied showed differential response ranging from tolerant to susceptible response to simulated drought stress. Different drought tolerance parameters are present in different inbreds that are classified as drought tolerant. There was great extent of variation in physiological parameters in the studied inbreds. This implied that there is possibility of identifying drought tolerant genotypes among the germplasm collections available with the breeder and could be used to produce drought tolerant hybrids as a short term breeding objective. Further, as a long term breeding objective, the drought tolerant corn inbreds CAL 1426–2, PDM 4641 and GPM 114 with different drought tolerance mechanisms (component traits) could be inter-mated to produce better drought tolerant composites which can further be subjected to recurrent selection approaches to derive inbred lines genetically enhanced for drought tolerance. Inter-mating to accumulate favourable alleles and to combine different mechanisms into a single inbred is necessary as single mechanism of tolerance will not suffice to obtain highly drought tolerant cultivars. The results of the present study further confirmed that the corn, a highly cross pollinated crop, presents enormous variability for most traits including drought tolerance.

## Supporting information

S1 Table Analysis of covariance between days to silking with physiological, phonological, yield and yield components under well watered condition. (DOCX)
