# Supplementary material for: Simulated drought stress unravels differential response and different mechanisms of drought tolerance in newly developed tropical field corn inbreds
Source: PLoS One. 2023 Mar 27;18(3):e0283528. doi: 10.1371/journal.pone.0283528 (PMC10042379; doi:10.1371/journal.pone.0283528)
Supplement: S1 Table — (DOCX) [file pone.0283528.s001.docx]

Supplementary Table: Analysis of covariance between days to silking with physiological, phonological, yield and yield components under well watered condition

| **Source of variation** | **df** | **RWC60** | **RWC75** | **SLW60** | **SLW75** | **SCMR60** | **SCMR75** | **Proline** | **Wax** | **Pollen**  **fertility** | **ASI** | **PH** | **EH** | **EL** |
| --- | --- | --- | --- | --- | --- | --- | --- | --- | --- | --- | --- | --- | --- | --- |
| Replication | 1 | -5.266 | -5.230 | 0.036 | -259.0 | -4.474 | -9.213 | -0.896 | -0.124 | 6.473 | -3.625 | 18.539 | 2.382 | -1.631 |
| Genotypes | 27 | 36.310 | 22.276** | -0.112** | -243.8 | 7.066 | 6.805 | -1.695** | -0.463 | -7.151 | -2.439 | 133.431** | 87.310 | 9.017 |
| Error | 27 | -0.066 | 0.028 | -0.002 | 0.001 | -0.277 | -0.629 | -0.052 | 0.031 | 0.0567 | 0.208 | 1.113 | -0.127 | -0.141 |

| **Source of variation** | **df** | **EG** | **KRN** | **NKR** | **SP** | **100GW** | **HI** | **GY** |
| --- | --- | --- | --- | --- | --- | --- | --- | --- |
| Replication | 1 | 0.859 | 2.900 | 1.036 | 2.046 | -5.696 | 4.453 | 0.264 |
| Genotypes | 27 | 1.809 | 3.685 | 3.505** | -2.449 | 6.368** | 0.562 | -3.142** |
| Error | 27 | -0.025 | -0.107 | 0.0766 | -0.226 | 0.099 | 0.508 | -0.33 |

** - indicates significant covariance at 1 % level of probability

RWC: Relative Water Content; SLW: Specific leaf weight; SCMR: SPAD chlorophyll meter reading; ASI: Anthesis silking interval PH: Plant height ; EH: Ear height; EL: Ear length; ED: Ear Diameter; KRN: Number of kernel rows; NKR: Number of kernels per row SP: Shelling percentage; 100GW: 100 grain weight; HI: Harvest Index; GY: Grain yield
